# Incidence of tuberculosis among HIV infected individuals on long term antiretroviral therapy in private healthcare sector in Pune, Western India

**DOI:** 10.1186/s12879-019-4361-0

**Published:** 2019-08-13

**Authors:** Ameet Dravid, Kartik Natarajan, Mahenderkumar Medisetty, Raviraj Gawali, Uma Mahajan, Milind Kulkarni, Chinmay Saraf, Charuta Ghanekar, Sachin Kore, Niranjan Rathod, Mrudula Dravid

**Affiliations:** 1 Department of Medicine, Ruby Hall Clinic, Pune, Maharashtra India; 2 Department of Medicine, Poona Hospital and Research Centre, Pune, Maharashtra India; 3 Department of Medicine, Noble hospital, Pune, Maharashtra India; 4 Department of Biostatistics, Precision Diagnostics and Biosciences, Pune, Maharashtra India; 5 Department of Pathology, Precision Diagnostics and Biosciences, Pune, Maharashtra India; 6 Department of Microbiology, Poona Hospital and Research Centre, Pune, Maharashtra India; 7 Department of Dermatology, Ashwini Sahakari Rugnalaya and Research Centre, Solapur, Maharashtra India; 8 Department of Medicine, Apex hospital, Kolhapur, Maharashtra India; 9 Department of Microbiology, Infectious Disease Clinic, Dhule, Maharashtra India

**Keywords:** Incident tuberculosis, HIV, India, Private healthcare sector, Antiretroviral therapy, Isoniazid preventive therapy

## Abstract

**Background:**

Despite rapid scale up of antiretroviral therapy (ART), Tuberculosis (TB) remains the commonest opportunistic infection and cause of death among HIV infected individuals in resource limited settings like India. Incidence of TB in individuals on ART in private healthcare sector in India is infrequently studied.

**Methods:**

This retrospective cohort study conducted between 1st March 2009 and 1st March 2017 aimed to evaluate rate of incident TB in individuals initiated on ART at 3 private sector ART clinics in Pune, India. Individuals more than 12 years of age with ART duration of atleast 6 months were included. Patients were classified as having prevalent TB if they had a TB episode within the year prior to ART initiation or if they developed TB within 6 months of starting ART. Individuals who were diagnosed with TB after 6 months of starting ART were classified as incident TB cases. A recurrent episode of TB after treatment completion or cure of prevalent TB was also regarded as incident TB. Patients were classified as definitive TB if *Mycobacterium tuberculosis* was grown in culture from a biological sample or a positive rapid molecular test. Patients were classified as probable TB if there was radiologic evidence of TB in absence of confirmatory culture or PCR.

**Results:**

1904 patients with a median duration of follow up on ART of 57 (IQR = 32.0, 84.0) months were included. Of these, 182 developed incident TB (22% definitive TB, 38% recurrent cases). TB incidence at 6–12 months, 13–24 months, 25–60 months and > 60 months of ART was 24.32, 5.46, 2.54 and 0.75 cases per 100 person years respectively. Current time updated CD4 count < 500 cells/mm^3^ (*p* < 0.0001), virologic failure on ART (adjusted Hazard Ratio (aHR): 3.05 (95% CI: 2.094, 4.454), *p* < 0.0001) and receipt of ART without IPT (aHR: 8.24 (95% CI, 3.358, 20.204), *p* < 0.0001) were associated with higher risk of incident TB.

**Conclusion:**

Starting ART early in treatment naïve individuals, prompt detection of virologic failure on ART and providing IPT along with ART will be useful in reducing incident TB. Efforts from private sector are crucial in achieving Sustainable Development Goals set by Government of India and attaining the vision of a TB free India.

## Background

Tuberculosis (TB) is a major global public health problem. In 2016, 10.4 million cases of TB were reported to World Health Organization (WHO) worldwide. India accounts for one fourth (2.8 million cases) of global TB burden [1]. Approximately 0.5 million Indians die annually because of TB [2, 3]. In addition, there are more than a million cases of TB each year which escape surveillance and are not notified to public health authorities in India. Majority of these individuals seek care in the private healthcare sector where they are not properly diagnosed and treated [2, 3]. HIV infection remains a known risk factor for development of TB and a key component for resurgence of incident TB in low and middle income countries like India [1, 4]. Approximately 5% of the incident TB cases in India have co-morbidity with HIV [2, 3]. An estimated 87,000 HIV associated TB cases were reported in India in 2016 and 12,000 patients died among them [2, 3].

Interventions to reduce HIV associated TB include antiretroviral therapy (ART), intensified TB case finding, isoniazid preventive therapy (IPT) and infection control. The use of ART reduces the risk of developing TB by 70–90% [5, 6]. As of 2017, 1.1 million individuals were on ART in India [7]. Although, addition of Isoniazid preventive therapy (IPT) to ART is shown to further reduce TB incidence [8], only 15% of eligible patients in India were on IPT as of 2017 [2, 3].

Modeling studies have suggested that expansion of ART coverage, improvement in TB diagnosis and treatment can help in drastic reduction in TB incidence and mortality [9]. However, cohort studies across regions do not share the same enthusiasm. They have shown high initial rates of TB in the first year of starting ART followed by gradual time-dependent reduction and stabilization above the estimated background population rate [10–24]. In addition, long term data on ART associated TB is limited. Median follow-up on ART in cohort studies rarely exceeds 2 years [4, 10, 11, 14, 20, 23]. As a result, calculation of long-term TB incidence rate is hampered due to small number of participants and events [20].

Incidence of TB among HIV infected individuals on ART in India is infrequently studied [25, 26], especially in the private healthcare sector which caters to almost 50% of overall TB cases [27]. Impact of ART related CD4 count recovery and virologic status on TB incidence has also not been well studied [25, 26]. Our study was aimed to calculate the rate of incident TB (incident TB > 6 months after starting ART) in HIV infected patients on ART in a private sector healthcare setting in Pune, Western India and to study risk factors associated with it.

## Methods

### Study setting

This retrospective cohort study was conducted at private sector ART clinics in three tertiary level hospitals (Ruby Hall Clinic, Poona Hospital and Noble Hospital) in Pune, Western India. Pune is located in state of Maharashtra which has the highest estimated number of people living with HIV (0.33 million, IQR - 0.25-0.43 million) in India [7] and has an annual TB notification rate of > 150 cases per 100,000 people per year [2, 3].

These three private hospitals provide clinical care, diagnostic and treatment services to HIV infected individuals in the state of Maharashtra at a subsidized cost. Patients are referred from primary care physicians, private practitioners of alternative medical systems, antenatal clinics and TB clinics. Patient data, including demographic, clinical, laboratory and treatment are entered into an electronic database (Livehealth software solutions, Pune, India).

### Data collection and patient characteristics

All patients > 12 years of age who had registered into the ART program between 1st March 2009 and 1st March 2017, initiated ART and completed atleast 6 months of follow up were included. Individuals who were non- naïve to ART at enrollment due to transfer in from another service were excluded. Baseline demographic data like age, gender, weight, hepatitis B co-infection, prevalent TB, co-morbidities like diabetes mellitus (DM), CD4 count (FACS Count, Becton Dickinson, Franklin Lakes, NJ, USA), addictions and hemoglobin (Hb) were collected from the database. CD4 count and Plasma HIV-1 viral load (NucliSENS Easy Q real-time nucleic acid sequence-based amplification (NASBA), BioMérieux®, France, detection limit - 20 to 10,000,000 copies/ml) values, which were done 6 months after ART start and yearly thereafter were also collected. Details of ART regimens and duration of ART was recorded for all patients. WHO and Indian National AIDS control organization (NACO) guidelines [28, 29] were followed for starting first line ART and switch to second line or third line ART in our cohort.

### Ethics approval and consent to participate

Access to raw data for clinical research was approved by Institutional review board (IRB) of all three hospitals (Ruby Hall Clinic, Poona Hospital and Noble Hospital).

### Tuberculosis screening and diagnosis

From March 2009 to March 2011, a symptom screen consisting of fever of any duration, cough for > 2 weeks, night sweats, weight loss of > 10%, chest pain, hemoptysis and no response to outpatient antibiotic therapy was used to investigate for TB at baseline and every follow up visit [30]. Since March 2011, WHO symptom score (weight loss, fever, night sweats, and cough of any duration) was used for TB screening as a standard of care at all 3 hospitals [8]. Individuals with positive symptom score were investigated by radiologic (X ray chest, ultrasound of abdomen or Computerized tomography (CT) scan of thorax, abdomen and pelvis) and/or laboratory (Ziehl Nelson staining of biological samples) methods. TB confirmation was done by automated liquid TB culture (Mycobacterial growth indicator tubes, MGIT 960, Becton Dickinson, Sparks, Maryland, USA) or Cartridge based Nucleic acid Amplification test (CB-NAAT, Xpert MTB/RIF, Cepheid, USA) or TB line probe assay (LPA, Hain Lifesciences, Germany). Patients were classified as definitive TB if *Mycobacterium tuberculosis* was grown in culture from a specimen taken from patient (sputum, pus, biopsied tissue, etc.) or a positive TB CB-NAAT or positive TB LPA. Patients were classified as probable TB if there was radiologic evidence of TB or evidence of acid-fast bacilli (AFB) on Zn stain in absence of confirmatory culture or PCR.

### Definition of prevalent TB and incident TB

Patients were classified as having prevalent TB if they had a TB episode within the year prior to ART initiation or if they already were on TB treatment when ART was started. Patients developing TB within 6 months of starting ART (early incident TB) were also classified as prevalent TB. Patients who had no history of prevalent TB and were diagnosed to have tuberculosis > 6 months after starting ART were classified as having incident TB. A recurrent episode of TB after treatment completion or cure of baseline prevalent TB was also regarded as incident TB. Upon diagnosis of prevalent or incident TB, TB type (Extrapulmonary (EPTB) or pulmonary (PTB)) [31], date of TB treatment initiation and completion and TB drug susceptibility testing (DST) results were recorded. All cases of TB were treated as per latest TB treatment guidelines [32, 33].

### Use of ART with IPT

After publication of 2011 WHO IPT guidelines [8], a subset of patients was initiated on IPT in addition to ART after excluding active TB. IPT was prescribed for 6 months irrespective of Tuberculin skin test (TST) status. Data of patients taking ART plus IPT as opposed to ART alone was recorded.

### Statistical methods

Continuous variables were summarized using median and interquartile range (IQR), while categorical variables were summarized using frequency and percentages. Continuous variables were compared using a median test. Categorical variables were compared using Chi-square test and Fishers’ exact test. TB incidence rate was defined as number of TB cases occurring per 100 person years after ART initiation. Duration of ART was calculated from date of ART initiation to development of first episode of incident TB, death of patient, lost to follow up (no clinic visits for more than 6 months) or censoring of observations on 1st March 2018. Person time accrued on ART during concomitant treatment of prevalent TB was excluded from denominator while calculating TB incidence rates.

Baseline and Time dependent risk factors associated with incident TB were identified by univariate and multivariate Cox proportional hazard model. Baseline risk factors considered were age (≤ 40 years or > 40 years), gender, prevalent TB, pre-ART CD4 count (CD4 ≤ 200 and >  200), addictions and hemoglobin (Hb < 10 g/dl or ≥ 10 g/dl). Time dependent risk factors included were current time updated CD4 count, virologic status on ART (virologically suppressed (VS) or virologic failure (VF)) and use of ART with IPT. All data was analyzed by STATA version 12.0.

## Results

**Fig. 1:**
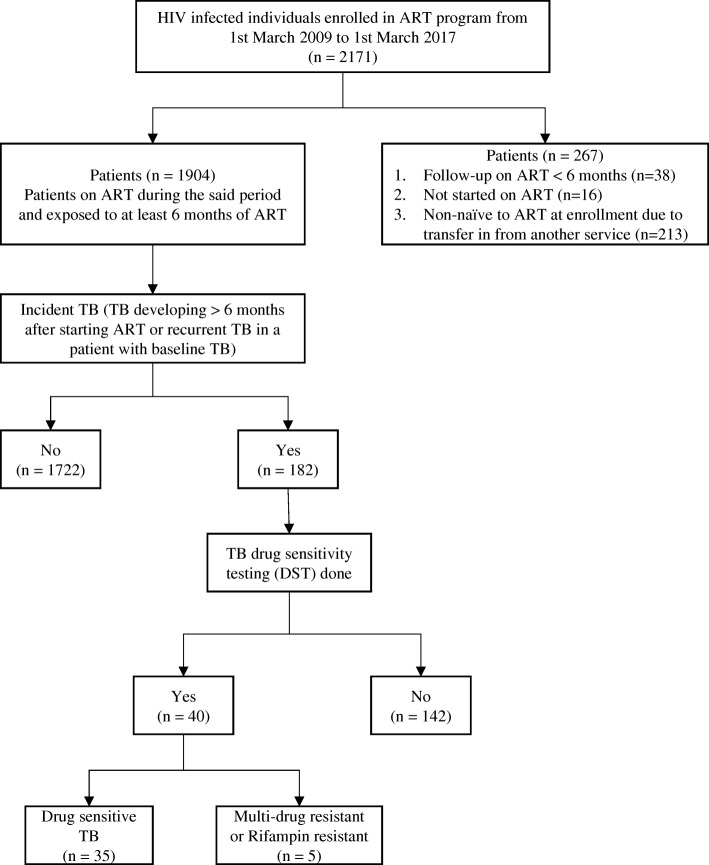
Flow chart depicting identification of individuals with Incident TB in Pune cohort

A total of 1904 HIV infected individuals (34.4% females) contributed to 9816.1 person years of follow up on ART (Fig. 1). Median age was 40 years (IQR = 33.0, 46.0) and median CD4 count prior to ART initiation was 173 (IQR = 83.0, 255.0) cells/mm^3^. CD4 count prior to ART initiation was less than 50 cells/mm^3^ in 14.1% patients. Hepatitis B surface antigen (HBsAg) was positive in 2.7% patients and 4.4% had baseline diabetes. Median duration of follow-up since HIV diagnosis was 62 (IQR = 36.0, 92.0) months and median duration of follow up on ART was 57 (IQR = 32.0, 84.0) months. One thousand four hundred and eighty-two (77.8%) and 413 (21.7%) patients were on first line and second line ART regimens respectively. First line ART regimens comprised of 2 nucleoside reverse transcriptase inhibitors (NRTI) plus 1 non-nucleoside reverse transcriptase inhibitor (NNRTI)). Second line ART regimens contained 2 NRTI plus 1 ritonavir boosted protease inhibitor (PI) or 1 boosted PI with 1 integrase inhibitor. Tenofovir with Emtricitabine was the most commonly used NRTI backbone. Efavirenz was the most common NNRTI and Ritonavir boosted Atazanavir (ATV/r) was the most common PI used in our cohort. Virologic suppression (VS) was seen in 84.4% patients (plasma viral load (pVL) < 1000 copies/ml) while virologic failure (VF, pVL >  1000 copies/ml) was seen in 15.6% patients at the time of latest plasma viral load estimation. At the end of the study period, 38.6% patients had reached a CD4 count threshold of > 500 cells/mm^3^. Of the total cohort, 18.8% (358/1904) were prescribed IPT with ART.

### Baseline prevalent TB

Baseline prevalent TB was seen amongst 33.5% (637/1904) patients in our cohort. Of these, 80.7% had EPTB while 19.3% had PTB. Male patients were 2 times more likely to have baseline TB than females (*p* < 0.0001). Median age of individuals with prevalent TB was 40 years (IQR = 34.0, 46.0) and median CD4 count at ART initiation was 108 (IQR = 58.0, 182.0) cells/mm^3^. Baseline prevalent TB was seen in 52, 12 and 6.3% patients with pre-ART CD4 counts ≤50, 51–200 and 201–500 cells/mm^3^ respectively. Patients with prevalent TB had higher tobacco (*p* < 0.0001) and alcohol addiction (*p* < 0.0001), lower Hb (*p* < 0.0001) and lower CD4 count at ART initiation (*p* < 0.0001) as compared to those without prevalent TB (Tables 1 and 2). Patients with prevalent TB had lower time updated CD4 count and higher incidence of VF on ART compared to patients without prevalent TB (*p* < 0.0001, Table 2).

**Table 1:** Baseline demographic data of patients in cohort

Characteristics	Total patients (*n* = 1904)	Prevalent TB at baseline		*p*-value
		No	Yes	
Total patients, *n* (%)	1904 (100%)	1267 (66.5%)	637 (33.5%)	
Age (years), Median (IQR) †	40 (33.0, 46.0)	40 (32.0, 47.0)	40 (34.0, 46.0)	0.491
Gender, *n* (%)				< 0.0001
Female	654 (34.4%)	507 (77.5%)	147 (22.5%)	
Male	1250 (65.6%)	760 (60.8%)	490 (39.2%)	
Ever Smoker, *n* (%)				0.067
Yes	221 (11.6%)	135 (61.1%)	86 (38.9%)	
No	1683 (88.4%)	1132 (67.3%)	551 (32.7%)	
Ever Tobacco user, *n* (%)				< 0.0001
Yes	840 (44.1%)	482 (57.4%)	358 (42.6%)	
No	1064 (55.9%)	785 (73.8%)	279 (26.2%)	
Ever consumed Alcohol, *n* (%)				< 0.0001
Yes	642 (33.7%)	376 (58.6%)	266 (41.4%)	
No	1262 (66.3%)	891 (70.6%)	371 (29.4%)	
Hepatitis B positive, *n* (%)				0.074
Yes	51 (2.7%)	28 (54.9%)	23 (45.1%)	
No	1853 (97.3%)	1239 (66.9%)	614 (33.1%)	
Prior Diabetes Mellitus, *n* (%)				0.093
Yes	84 (4.4%)	63 (75.0%)	21 (25.0%)	
No	1820 (95.6%)	1204 (66.2%)	616 (33.9%)	
Haemoglobin at baseline (g/dl) (*n* = 1592)^a^, *n* (%)				< 0.0001
≤ 10	388 (24.4%)	173 (44.6%)	215 (55.4%)	
> 10	1204 (75.6%)	839 (69.7%)	365 (30.3%)	

*IQR* Interquartile range, *TB* tuberculosis. ^a^Hemoglobin data is not available for 312 patients

**Table 2:** Antiretroviral therapy data of patients in cohort

Characteristics	Total patients (*n* = 1904)	Prevalent TB at baseline		*p*-value
		No	Yes	
Total patients, *n* (%)	1904 (100%)	1267 (66.5%)	637 (33.5%)	
ART regimes (*n* = 1894)^b^, *n* (%)				0.947
NNRTI based	1482 (78.2%)	983 (66.3%)	499 (33.7%)	
PI based	413 (21.8%)	275 (66.5%)	138 (33.5%)	
Duration since HIV diagnosis (Months), Median (IQR) ^a^	62 (36.0, 92.0)	61 (34.0, 92.0)	64 (36.0, 94.0)	0.231
Duration on ART (Months), Median (IQR)^a^	57 (32.0, 84.0)	56 (32.0, 85.0)	60 (32.0, 82.0)	0.382
CD4 count at ART initiation, Median (IQR) ^a^	173 (83.0, 255.0)	197 (112.0, 297.0)	108 (58.0, 182.0)	< 0.0001
CD4 count NADIR, Median (IQR) ^a^	146 (72.0, 235.0)	183 (88.0, 277.0)	88 (49.0, 165.0)	< 0.0001
Current/updated CD4 count, Median (IQR) ^a^	402 (236, 620)	440 (286, 664)	331 (187, 521)	< 0.0001
Virologic condition on ART, *n* (%)				< 0.0001
Virologic failure (VF)	297 (15.6%)	166 (55.9%)	131 (44.1%)	
Virologic suppression (VS)	1607 (84.4%)	1101 (68.5%)	506 (31.5%)	
IPT on ART, *n* (%)				0.001
ART plus IPT given	358 (18.8%)	264 (73.7%)	94 (26.3%)	
ART alone	1546 (81.2%)	1003 (64.9%)	543 (35.1%)	

*ART* antiretroviral therapy, *IPT* ionized preventive therapy, *IQR* interquartile range, *NNRTI* nonnucleoside reverse transcriptase inhibitor, *PI* protease inhibitor, *TB* tuberculosis. ^a^ Median test, ^b^Integrase inhibitor based ART regimen was given to 10 patients

Incident TB was detected in 9.6% (182/1904) patients (27.5% females). Of the total incident cases, 73.9% patients had EPTB while 26.1% had PTB. Median age of individuals developing incident TB was 39 yrs. (IQR = 33.0, 46.0) and median CD4 count at ART initiation was 142 (IQR = 78.0, 198.0) cells/mm^3^. Median CD4 count at the time of incident TB diagnosis was 156 (IQR = 68.0, 314.0) cells/mm^3^. Incidence rate for TB was 1.85 cases (95% CI: 1.604–2.144) per 100 person years. Median time to development of incident TB was 39 (IQR = 16.0, 68.0) months. Of all the patients with incident TB, 69 (37.9%) had a history of prevalent TB. Median time interval between end of prevalent TB and diagnosis of incident TB was 38 months (IQR = 16.0, 66.0). The rate of incident TB was higher in patients with prevalent TB as compared to those without prevalent TB (2.11 (95% CI: 1.666–2.671) and 1.73 episodes (95% CI: 1.436–2.076) per 100 person years respectively, *p* = 0.181, Table 3).

**Table 3:** Rate of Incident TB in entire cohort and subgroup analysis

Characteristics	Entire Cohort (*n* = 1904)		Patients with NO baseline prevalent TB (*n* = 1267)	
	Number of patients (n)	Incidence rate (95% CI)per 100 person years	Number of patients (n)	Incidence rate (95% CI) per 100 person years
Total patients	182	1.85 (1.604, 2.144)	113	1.73 (1.436, 2.076)
Age (years)				
≤ 40 years	100	1.83 (1.502, 2.223)	63	1.75 (1.363, 2.233)
> 40 years	82	1.89 (1.521, 2.344)	50	1.71 (1.292, 2.249)
Gender				
Female	50	1.49 (1.132, 1.971)	35	1.34 (0.961, 1.865)
Male	132	2.04 (1.721, 2.421)	78	1.98 (1.589, 2.478)
History of prior TB at baseline				
Yes	69	2.11 (1.666, 2.671)		
No	113	1.73 (1.436, 2.076)		
Haemoglobin at baseline (g/dl)	(*n* = 156)		(*n* = 88)	
≤ 10	53	2.84 (2.171, 3.719)	65	2.79 (1.853, 4.196)
> 10	103	1.58 (1.304, 1.919)	23	1.43 (1.125, 1.830)
Virologic status on ART				
Virologic failure (VF)	105	8.51 (7.030, 10.307)	71	10.23 (8.107, 12.910)
Virologic suppression (VS)	77	0.90 (0.717, 1.122)	42	0.72 (0.530, 0.972)
IPT				
ART plus IPT	5	0.20 (0.081, 0.469)	2	0.11 (0.026, 0.423)
ART alone	177	2.44 (2.106, 2.828)	111	2.38 (1.979, 2.872)
Duration of ART (months)				
≤ 12	33	24.32 (17.293, 34.215)	23	25.58 (16.998, 38.492)
13–36	52	5.46 (4.162, 7.167)	29	4.32 (3.005, 6.223)
37–60	44	2.54 (1.890, 3.413)	28	2.41 (1.666, 3.495)
> 60	53	0.75 (0.579, 0.992)	33	0.72 (0.507, 1.004)
Pre ART CD4 count (cells/mm3)				
≤ 50	32	2.28 (1.612, 3.224)	18	2.57 (1.621, 4.085)
51–200	108	2.20 (1.824, 2.660)	59	1.98 (1.537, 2.560)
201–500	41	1.28 (0.943, 1.740)	35	1.35 (0.967, 1.875)
> 500	1	0.32 (0.045, 2.303)	1	0.36 (0.052, 2.625)
Current/ updated CD4 count (cells/mm3)				
≤ 200	107	7.36 (6.090, 8.896)	65	8.35 (6.548, 10.648)
201–350	36	1.75 (1.265, 2.432)	22	1.64 (1.078, 2.486)
351–500	20	1.03 (0.667, 1.602)	12	0.97 (0.552, 1.711)
> 500	19	0.43 (0.277, 0.681)	14	0.44 (0.260, 0.741)

*ART* antiretroviral therapy, *IPT* Isoniazid preventive therapy, *TB* tuberculosis

Of the incident TB cases, 21.9% (40/182) patients had culture or CB-NAAT confirmed TB while 78.1% (142/182) had probable TB. Thirty-three (33/182, 18.1%) patients developed incident TB between 6 and 12 months of starting ART while 142 (81.9%) developed it after 12 months of starting ART. Seventy-seven (42.3%) patients with incident TB were virologically suppressed on ART while 105 (57.7%) were virologic failures. Out of the 40 patients with definite TB, 35 had drug sensitive TB while 5 had Multi-drug resistant TB or Rifampicin mono-resistant TB on LPA. None of our patients who were subjected to LPA showed Isoniazid mono-resistant TB.

### ART duration and rate of incident TB

TB incidence rate at 6–12 months, 13–24 months and 25–60 months of ART was 24.32, 5.46 and 2.54 cases per 100 person years respectively (Table 3). After 5 years on ART, there was a reduction in TB incidence rate to 0.75 cases per 100 person years.

Risk factors like age (*p* = 0.668), gender (*p* = 0.068) and baseline prevalent TB (*p* = 0.181) were not associated with incident TB. Baseline factors like CD4 count ≤200 cells/mm^3^ (*p* < 0.0001), Hb ≤ 10 g/dl (*p* = 0.001) and history of chewing tobacco (*p* < 0.0001) or consuming alcohol (*p* = 0.028) were associated with higher risk of incident TB on univariate analysis only (Table 4). Current time updated CD4 count < 500 cells/mm^3^ (*p* < 0.0001), virologic failure on ART (aHR: 3.05 (95% CI: 2.094, 4.454), *p* < 0.0001) and receipt of ART without IPT (aHR: 8.24 (95% CI: 3.358, 20.204), *p* < 0.0001) were associated with higher risk of incident TB on univariate and multivariate Cox regression analysis (Table 4).

**Table 4:** Cox Proportional Hazard Model for identifying risk factors for incident TB

Characteristics	Univariate analysis		Multivariate analysis	
	Hazard ratio (95% CI)	*p*-value	Adjusted Hazard ratio (95% CI)	*p*-value
Age > 40 years (Ref: ≤ 40 years)	1.07 (0.794, 1.432)	0.668	1.01 (0.746, 1.375)	0.935
Male (Ref: Female)	1.36 (0.978, 1.877)	0.068	0.96 (0.616, 1.495)	0.855
Ever Tobacco user (Ref: not used tobacco)	1.90 (1.413, 2.560)	< 0.0001	1.08 (0.737, 1.584)	0.690
Ever consumed Alcohol (Ref: not consume alcohol)	1.39 (1.036, 1.866)	0.028	1.10 (0.770, 1.572)	0.599
History of baseline prevalent TB (Ref: No baseline prevalent TB)	1.23 (0.909, 1.656)	0.181		
Haemoglobin at baseline ≤10 g/dl (*n* = 1592)(Ref: > 10 g/dl)^a^	1.80 (1.290, 2.504)	0.001		
Virologic failure on ART (VF)(Ref: Virologic success (VS))	9.51 (7.078, 12.786)	< 0.0001	3.05 (2.094, 4.454)	< 0.0001
ART alone (Ref: ART plus IPT)	12.71 (5.217, 30.943)	< 0.0001	8.24 (3.358, 20.204)	< 0.0001
Pre ART CD4+ ≤ 200 cells/mm3(Ref: > 200 cells/mm3)	1.87 (1.326, 2.647)	< 0.0001	1.18 (0.816, 1.714)	0.377
Pre ART CD4+ (Ref: > 500 cells/mm3)				
≤ 50	6.87 (0.937, 50.321)	0.058		
51–200	6.60 (0.921, 47.370)	0.060		
201–500	3.79 (0.521, 27.596)	0.188		
Current/updated CD4+ (Ref: > 500 cells/mm3)				
≤ 200	17.60 (10.783, 28.716)	< 0.0001	6.62 (3.678, 11.915)	< 0.0001
201–350	4.19 (2.401, 7.313)	< 0.0001	3.22 (1.808, 5.746)	< 0.0001
351–500	2.43 (1.297, 4.555)	< 0.0001	2.05 (1.083, 3.888)	0.027

*ART* antiretroviral therapy, *IPT* isoniazid preventive therapy, *TB* tuberculosis

^a^Hemoglobin data is not available for 312 patients, hence excluded from the multivariate analysis

## Discussion

India’s vast, heterogeneous and unregulated private sector is plagued by poor diagnostic practices for TB leading to diagnostic delay, inadequate TB treatment regimens due to prescription errors, high rates of treatment non-completion due to poor adherence counseling and low notification rates to public health authorities [27, 34–36]. Despite these drawbacks, private healthcare sector caters to almost 50% of overall TB cases in India (private-sector average tuberculosis burden of 2.2 million cases in 2014) [27] and TB incidence studies in private sector are long overdue. To the best of our knowledge, this is the first report on incident TB amongst ART experienced HIV infected individuals from Indian private healthcare sector. All patients were attended to by qualified allopathic healthcare providers at all 3 hospitals. In our cohort, we observed a 9.6% 5 year risk of developing incident TB on ART, compared to the 10% lifetime risk in HIV uninfected individuals [37].

### Comparison with TB incidence studies from India

We found 2 studies which reported the occurrence of ART associated TB from the Indian public health sector with a follow-up period of 6 months to 4 years [25, 26]. These studies observed an incidence rate of 2.4–2.83 cases per 100 person years for the entire follow-up period which is higher than that seen in our study (1.85 cases per 100 person years). Lower incidence of TB in our cohort compared to the 2 reported studies could be due to the availability of better preventive, diagnostic and treatment set-ups and strategies for controlling TB [23]. Lower incidence rate of TB could also be due to longer follow up on ART in our cohort. Drawbacks of these studies [25, 26] include limited follow up on ART, majority of incident TB cases occurring in first 6 months post ART, all patients with symptoms not actively investigated for TB, non-availability of time updated CD4 count and viral load values and nonuse of culture or molecular methods for TB diagnosis. The strengths of our cohort include its large size, maintenance of electronic records of all patients, prolonged duration of follow-up (median of 5 years), annual CD4 count and pVL monitoring, intensive TB case finding by applying WHO symptom score at every clinic visit, data on use of IPT and ART and recording of treatment outcomes of patients on antitubercular therapy (ATT).

### Prevalent TB

We demonstrated a baseline TB prevalence rate of 33.5% among patients initiating ART in our cohort which is higher than previous reports from other resource-limited settings [4, 12, 38]. Higher prevalence could be related to intensive TB case finding at baseline or due to inclusion of patients with early incident TB as prevalent TB. This was done to eliminate the bias which might arise if prevalent TB is erroneously recorded as early incident TB due to limitation of available diagnostic tests. Rarely, TB may be missed during baseline screening and patients are started on ART. They may develop unmasking TB immune reconstitution inflammatory syndrome (IRIS) within 3 months of starting ART [39] and be falsely counted as incident TB. Diagnosing asymptomatic or subclinical TB among treatment naïve HIV infected patients is indeed complex and challenging [40]. In our cohort, there was a higher prevalence of baseline TB among male patients. This might be due to higher prevalence of advanced HIV disease among males as compared to female patients (Baseline CD4 < 200 cells/mm^3^, 66.74% versus 50.38%). This finding was also seen in a recently published meta-analysis where males were found to be at 73% higher risk of advanced HIV disease at first clinic visit as compared to females [41]. Prevalent TB was also more common among patients with pre-ART CD4+ count < 50 cells/mm^3^ and anemia at baseline.

### Baseline CD4 count and incident TB

Severity of immune suppression at the time of ART initiation was associated with risk of incident TB on univariate Cox regression analysis. Compared to those starting ART at CD4 count > 200 cells/mm^3^, patients with low pre-ART CD4 count (≤ 200 cells/mm^3^) were almost twice as likely to develop incident TB (aHR: 1.87 (95% CI: 1.326–2.647). Those patients starting ART at CD4 >  500 cells/mm^3^ had the lowest risk of incident TB (0.32 cases per 100 person years). These findings were similar to that seen in study conducted by Bock et al. [42] in which TB incidence after ART initiation was significantly lower among individuals starting ART at CD4 counts above 500 cells/mL.

### Time updated CD4 count and incident TB

**Fig. 2:**
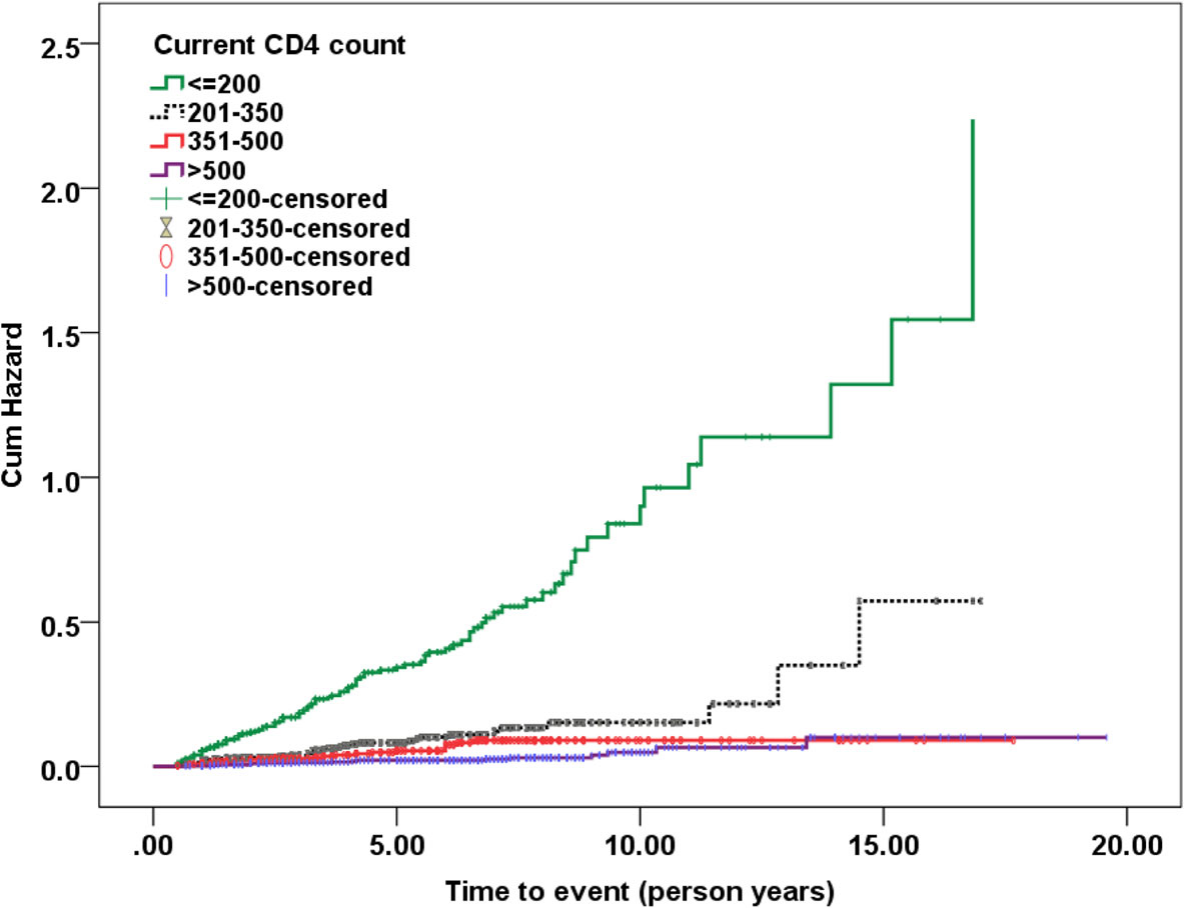
Kaplan-Meier estimates for Incident TB by Current time updated CD4 count

In addition, time updated CD4 count was associated with risk of incident TB on univariate and multivariate Cox regression analysis. Compared to patients with current time updated CD4 count > 500 cells/mm^3^, patients with current CD4 count ≤200, 201–350, and 351–500 cells/mm^3^ were six times (aHR: 6.62 (95% CI: 3.678, 11.915), *p* < 0.0001), three times (aHR: 3.22 (95% CI: 1.808, 5.746), *p* < 0.0001) and two times (aHR: 2.05 (95% CI: 1.083, 3.888), *p* = 0.027) more likely to develop incident TB respectively (Fig. 2). These findings were similar to those published by Van Rie et al. [4] and Lawn et al. [11] in which low time updated CD4 count was a risk factor for incident TB. After starting ART, CD4 count threshold of 500 cells/mm^3^ has to be exceeded to minimize incident TB risk [11]. In our cohort, despite effective ART almost 60% of individuals did not achieve this threshold. As a result, TB specific immune response was not properly reconstituted, and patients remained at risk of incident TB. These findings further strengthen the recommendation by current ART guidelines [28, 29] to start ART early and attain near normal CD4 count.

### Virologic failure on ART and incident TB

In our cohort, patients with virologic failure (VF) on ART (pVL >  1000 copies/ml) were 3 times more likely to develop incident TB (aHR: 3.05 (95% CI: 2.094, 4.454). This association is also seen in other cohort studies [20]. HIV replication despite effective ART leads to immune activation, increased T and B cell turnover, skewing of lymphocytes towards differentiated phenotypes and increased cellular senescence. In addition, regenerative capacity of immune cells is damaged. This leads to poor quality of humoral and cellular immune response to common antigens and increased susceptibility to opportunistic infections (OI) [43]. In the study by Kaplan et al., patients on ART having pVL > 150,000 copies/ml had 3 times higher risk of OI compared to patients with suppressed HIV replication (pVL < 400 copies/ml) [44]. Amongst ART experienced patients in South Africa, those with pVL > 10,000 copies/ml had a 41% greater risk of incident TB as compared to patients with pVL < 1000 copies/ml [45]. These findings emphasize the need for routine viral load testing for all patients on ART in India as per WHO [46] and NACO guidelines [47]. Early identification of virologic failure and immediate switch to fully active antiretroviral therapy can achieve viral re-suppression and reduce incidence of TB.

### IPT and incident TB

According to WHO, IPT should be administered to all HIV infected individuals residing in moderate to high TB incidence settings irrespective of their Tuberculin skin test (TST) status, once active TB is ruled out. IPT reduces the risk of developing incident TB by 33–67% for up to 48 months in such patients [48, 49]. In a randomized, double blind placebo-controlled trial conducted by Rangaka et al., 12 months of IPT independently reduced the incidence of TB among patients concurrently on ART by 37% [50]. Multiple cohort studies have also demonstrated the efficacy of ART plus IPT in preventing incident TB [51–53]. In our study, patients on ART alone were 8 times more likely to develop incident TB than patients on ART plus IPT (aHR: 8.24 (95% CI: 3.358, 20.204). The rate of incident TB was 0.2 cases/100 person years and 2.44 cases/100 person years among patients exposed to ART plus IPT and ART alone respectively. Our data clearly suggests that IPT and ART have additive efficacy in preventing incident TB. Out of the 358 patients exposed to ART plus IPT, only 5 (1.39%) developed incident TB. All episodes of incident TB occurred after discontinuation of IPT suggesting exogenous re-infection. The BOTUSA study comparing six-months versus 36-months of IPT in Botswana reported a 43% reduction in TB incidence with the longer regimen, an effect which was more striking among TST-positive individuals. The efficacy of 6 month regimen seemed to wane about 200 days after IPT discontinuation. [54]. In another randomized controlled trial from India, TB incidence was lowered by 6 month Isoniazid plus ethambutol (6HE) based preventive regimen and 36 month IPT regimen by 65 and 78% respectively. TB incidence was 40% lower with longer regimen, but difference was not statistically significant [55]. Our findings support the use of longer term IPT in individuals on ART in India to prevent both, reactivation of latent TB and exogenous reinfection.

### ART duration and incident TB

TB incidence rates in our cohort reduced from 24.32 cases per 100 person years at 6–12 months of ART to 0.75 cases per 100 person years after 5 years on ART. Decline in TB incidence may be related to the immune reconstitution and virologic suppression due to ART. Prior to starting ART, 61.1 and 4.5% patients had CD4 count < 200 and >  500 cells/mm^3^ respectively. After starting ART, only 18.95% patients had CD4 count < 200 cells/mm^3^ at study closure, while 38.9% patients had achieved CD4 count > 500 cells/mm^3^. Almost 85% patients had achieved virologic suppression. These findings were similar to that seen in other cohort studies from India [56]. Despite this reduction, TB incidence rates after 5 years of ART were higher than estimated community TB rate (150 cases per 100,000 population) [2, 3]. We think that this phenomenon of high TB incidence may be due to multiple factors which include inability to reach a CD4 count threshold of > 500 cells/mm^3^ despite long term ART, impaired restoration of TB specific immunity [57], low usage of IPT in our cohort and high rate of TB re-infection [58, 59]. The incidence rates observed are most likely due to a combination of these factors.

### Limitations of the study

Our study has several limitations. First, as for all retrospective studies, some episodes of TB may be unreported leading to measurement bias and underestimation of prevalent and incident TB. Second, Body mass index (BMI) was not recorded in all patients in our cohort. Poor nutritional status (BMI < 25 kg/m^2^) is a strong risk factor for incident TB [4]. Every unit increase in BMI leads to 13.8% reduction in TB incidence [60]. Third, person time spent in different time updated CD4 count strata and its association with risk of incident TB [20] was not calculated in our study. Fourth, in case of recurrent TB, we were unable to distinguish between recurrence due to exogenous re-infection or endogenous reactivation (relapse). Fifth, TB confirmation and Drug susceptibility testing (DST) by CB-NAAT or LPA was ascertained in only 22% of incident TB cases. The main reason for the same was high prevalence of EPTB in our cohort. DST like CB-NAAT and LPA suffer from lower sensitivity in extrapulmonary samples [61]. In addition, widespread availability of DST in India started only in 2017 [3]. We believe that these numbers will definitely improve in the future. Authors also agree that a minority of these patients diagnosed with probable TB could be actually suffering from Non tuberculous Mycobacterial (NTM) infection which could present with similar radiologic features [62]. We estimate that the “non cases” or NTM infections would be < 5% of identified incident TB cases. Sixth, outcome data after initiation of TB treatment was not always readily available for complicated patients referred for hospitalization or for those that were lost-to-follow-up.

## Conclusions

Our study from the Indian private healthcare sector has shown a high rate of incident TB in patients on ART. It clearly demonstrates that despite ART scale-up and improving life expectancy of HIV positive patients in India, sizeable proportion of individuals remain susceptible to incident TB and will fall prey to it unless obstacles to TB prevention are removed. Starting ART early in treatment naïve individuals, close monitoring for incident TB in patients with low baseline and time updated CD4 count, routine virologic monitoring of all patients on ART and routine use of ART with IPT are important takeaways from our cohort study. Efforts from private sector are crucial in achieving the Sustainable Development Goals set by Government of India and attaining the vision of a TB free India.

## Data Availability

The datasets used and/or analyzed during the current study available from the corresponding author on reasonable request.

## Abbreviations

aHR: adjusted Hazard Ratio
ART: Antiretroviral therapy
BMI: Body mass index
CB-NAAT: Cartridge based nucleic acid testing
CI: Confidence interval
DM: Diabetes mellitus
EPTB: Extrapulmonary tuberculosis
IPT: Isoniazid preventive therapy
IQR: Interquartile range
IRIS: Immune reconstitution inflammatory syndrome
LPA: Line probe assay
NACO: National AIDS control organization
NNRTI: Non- nucleoside reverse transcriptase inhibitor
NRTI: Nucleoside reverse transcriptase inhibitor
PI: Protease inhibitor
PTB: Pulmonary tuberculosis
pVL: Plasma viral load
TB: Tuberculosis
VF: Virologic failure
VS: Virologic suppression
WHO: World health organization
